# Gestational Exposure to Low Dose Bisphenol A Alters Social Behavior in Juvenile Mice

**DOI:** 10.1371/journal.pone.0025448

**Published:** 2011-09-28

**Authors:** Jennifer T. Wolstenholme, Julia A. Taylor, Savera R. J. Shetty, Michelle Edwards, Jessica J. Connelly, Emilie F. Rissman

**Affiliations:** 1 Department of Biochemistry and Molecular Genetics, University of Virginia School of Medicine, Charlottesville, Virginia, United States of America; 2 Division of Biological Sciences, University of Missouri, Columbia, Missouri, United States of America; 3 Cardiovascular Medicine, University of Virginia School of Medicine, Charlottesville, Virginia, United States of America; Università di Parma, Italy

## Abstract

Bisphenol A (BPA) is a man-made compound used to make polycarbonate plastics and epoxy resins; public health concerns have been fueled by findings that BPA exposure can reduce sex differences in brain and some behaviors. We asked if a low BPA dose, within the range measured in humans, ingested during pregnancy, would affect social behaviors in prepubertal mice. We noted sex differences in social interactions whereby females spent more time sitting side-by-side, while males engaged in more exploring and sitting alone. In addition BPA increased display of nose-to-nose contacts, play solicitations and approaches in both sexes. Interactions between sex and diet were found for self grooming, social interactions while sitting side-by-side and following the other mouse. In all these cases interactions were produced by differences between control and BPA females. We examined brains from embryos during late gestation to determine if gene expression differences might be correlated with some of the sexually dimorphic or BPA affected behaviors we observed. Because BPA treatments ended at birth we took the brains during embryogenesis to increase the probability of discovering BPA mediated effects. We also selected this embryonic age (E18.5) because it coincides with the onset of sexual differentiation of the brain. Interestingly, mRNA for the glutamate transporter, *Slc1a1*, was enhanced by exposure to BPA in female brains. Also we noted that BPA changed the expression of two of the three DNA methyltransferase genes, *Dnmt1* and *Dnmt3a.* We propose that BPA affects DNA methylation of *Sc1a1* during neural development. Sex differences in juvenile social interactions are affected by BPA and in particular this compound modifies behavior in females.

## Introduction

Bisphenol A (BPA) is a synthetic monomer used to manufacture polycarbonate plastics (i.e. food and water containers) and epoxy resins (i.e. canned food linings). Exposure to this chemical is fairly ubiquitous; as it has been detected in urine in 90% of all humans sampled [1]. BPA is detectable in maternal and fetal plasma in ranges from 0.3 to 18.9 and 0 to 9.2 ng/ml respectively [2]. Several countries have banned BPA in production of new products based on findings from animal studies that suggest BPA can affect the development of prostate, brain and behavior [3], [4], [5], [6], [7]. Neonatal treatment with BPA in general reduces sex differences in the brain and can modify neurite and dendrite formation [3], [8], [9], [10], [11]. Pre- and/or peri-natal exposure to BPA in rodents is associated with cognitive impairments and decreased exploration in a novel environment [12], [13], [14]. BPA can also influence social interactions and anxiety in rodents [7], [15], [16], [17]. In cynomolgus monkeys, prenatal BPA exposure in male offspring is correlated with increased outward looking and exploration, and it affects behavior of the mothers [18]. In humans, a positive association between gestational levels of BPA in mothers and externalizing (hyperactivity and aggression) behaviors in 2 year old girls has been reported [19]. This convergence of data demonstrates that BPA exposure during gestation affects the brain and a number of behaviors in several mammalian species.

Steroid hormones organize sex differences in the brain during neonatal development [20], [21]. BPA has steroid-like properties and binds to both estrogen receptors (ERα, ERβ) with low affinity [22], [23]. It also binds the estrogen membrane receptor (GPER) with high affinity [23] as well as androgen and thyroid receptors [24], [25], [26], [27]. Many authors have suggested that BPA exposure disrupts sexually dimorphic brain development and behaviors via its actions on the steroid receptors [9], [11], [28]. In addition to steroid-related effects, BPA may have even more global actions as it can act to alter DNA methylation [29]. Dysregulation of DNA methylation during these critical developmental windows could disrupt the normal progression of brain and endocrine system development causing robust changes in the developing embryo that can persist into adulthood or even beyond if effects extend to germ cells [30]. In addition, these two mechanisms may act synergistically, as DNA methyltransferases have been shown to have a number of interactions with estrogen receptors, particularly with ER ß [31].

In this set of studies we exposed female mice to a low dose of BPA mixed into food pellets. We validated our dose by measuring BPA concentrations in blood from pregnant dams consuming the diet. We assessed social interactions in juveniles gestated on control or BPA containing diets. We are interested in this period because, in humans it is a time when many neurodevelopmental disorders that have a social behavior component, are first detected. Moreover, many such disorders are skewed in their expression toward males, one well known example is Autism Spectrum Disorder (ASD) which is four times more prevalent in boys than in girls [32]. Because sexual differentiation of the hypothalamus begins in late gestation, we collected embryos on embryonic day 18.5 (E18.5) to assess differences in gene expression of candidate genes in control versus BPA exposed brains. We assessed gene expression with qPCR for the known estrogen receptors, DNA methyltransferases, and several genes related to glutamate and GABA transmission that have been identified as potential BPA targets in other studies [33]. Finally we examined mRNA for the oxytocin receptor because it has been implicated in a variety of social behaviors [34], [35].

## Methods

### Animals

All procedures were conducted in compliance with the University of Virginia Animal Use and Care Committee and in strict accordance with the recommendations in the Guide for the Care and Use of Laboratory Animals of the National Institutes of Health. All mice were housed on a 12∶12 light (lights off at 1300 EST). Adult female C57BL/6J (B6) mice were randomly assigned to one of two groups and placed on either a phytoestrogen-free chow (n = 11; Harlan Teklad, TD95092) or the same chow supplemented with 1.25 mg BPA per kg diet (n = 12; Harlan Teklad, TD09710). All females consumed their assigned diets (food and water) ad libitum. Over the last 10 days of gestation dams ingest about 4 grams daily of this type of chow [16]. At this dose of BPA we calculate intake to be roughly 5 µg of BPA daily. The US EPA lowest observed adverse effect level (LOAEL) for humans is 50 mg/kg/day [36]. To determine the maximum concentration believed to be safe, even for daily exposure, the EPA divides this dose by a 1,000-fold safety factor (50 µg/kg/day). Thus, a 150 pound (68 kg) pregnant woman could “safely” consume 3.4 mg BPA per day and our dose is 680 times lower than the LOAEL.

For embryo collection females were placed on one of the two diets as above. After one week on the diets, males were added to each cage with a single female, and every morning we checked for plugs. The day a plug was discovered was designated E0.5 and when a plug was noted the male cage mate was removed. Pregnant females were collected in the morning on E18.5, rapidly sacrificed with isoflurane and embryos extracted. Embryo brains were collected quickly and frozen on dry ice. Embryos were sexed by PCR [37] and we limited our use to embryos positioned *in utero* next to at least one male littermate to reduce any potential variation caused by intrauterine position [38]. We collected brains from 10 litters for use in this experiment (BPA n = 5, Control n = 5).

For the behavioral studies females were placed on one of the two diets and paired with a male as described above. Males remained with the females for seven days. Within 12 hours after birth all pups (from control and BPA consuming dams) were fostered to another dam that had delivered pups within the past 24 hours and was consuming the control diet. We did this to limit offspring exposure to BPA to the gestational period and because differences in maternal behavior caused by BPA might affect behavior [16], [39]. Foster dams (n = 20) had mixed litters of their biological, same-age pups (not in the study), and fostered pups. For identification purposes we randomly clipped tail tips of either the biological or foster pups at the time of fostering. All pups (BPA: n = 39; 18 females and 21 males, control: n = 28; 13 females and 15 males) remained with their dams until post natal (PN) 21, at which time they were group housed by litter and sex and tested for behaviors (see below).

### BPA assay

Pregnant dams, on gestation day 18.5, consuming control (n = 3) or BPA-supplemented diet (n = 4) were anesthetized with isoflurane and blood was collected by cardiac puncture, spun and serum frozen. Pooled sets of serum samples (∼0.3–0.8 ml) were aliquoted into glass tubes and spiked with an internal standard and extracted twice with methyl tert-butyl ether (Fisher Scientific, Pittsburgh, PA). The ether extracts were dried in glass tubes under nitrogen and reconstituted in 60∶40 methanol:water. Unconjugated (free) BPA was measured by HPLC with an ESA CoulArray 5600 detector. Separation was performed on a reverse-phase 250 mm Prodigy C18 column (Phenomenex), with a mobile phase of 36∶24∶40 acetonitrile: methanol: 0.05 M sodium acetate buffer (pH 4.8), and with the CoulArray cell potentials set at 325, 400, 720 and 875 mV. Bisphenol A (Sigma, St. Louis, MO) was used as the internal standard. Quantitation was made against standard curves of both analytes, and extraction efficiency was assessed from recovery of the internal standard, which averaged over 95%. The limit of detection (LOD) for BPA in serum by these methods was 0.5 ng/ml based on extraction of 0.5 ml of serum, and values below this level were estimated by extrapolation of the standard curve to zero. BPA was not detected by HPLC in either the assay blanks or in the solvent blanks used in the standard curve, nor in the tubes used to collect blood and store serum. Solvents and water used in the assay were HPLC grade, and previously tested negative for BPA. Use of plastic in the assay was limited to pipet tips (previously established not to leach BPA).

### Behavior Tests

Habituation and behavior tests were conducted in the dark (between 1300 and 1800 hours) under red light. Behaviors were recorded and later scored by an observer blind to sex and treatment group.

#### Juvenile social interactions

On PN20, the day before weaning, mice were singly housed in a novel standard mouse cage, with bedding but no food or water, for 1 hour to habituate to this novel environment. After habituation, mice were returned to their home cages with their siblings and dam. On PN21 mice were again habituated to the test room in a clean standard mouse cage for 1 hour then placed into another clean cage with a same age, sex and treatment mouse from another litter. Social interactions were recorded for both mice for 30 minutes. Mice were evaluated for a number of social and non-social behaviors using Noldus Observer (5.0) software (Noldus, Leesburg, VA, USA). Details on our methods and scoring are published [40], [41]. The social behavior categories included total amount of time spent displaying; side-by-side sitting, grooming their partners or engaged in side-by-side non-grooming interactions (including digging or manipulating bedding). Non-social behaviors consisted of time spent exploring the cage, self-grooming, and sitting alone. Some behaviors are of short duration, and thus it is more appropriate to score their frequencies. The frequencies of interactive behaviors we recorded included acts of nose-to-nose or anogenital sniffing, crawling over or under the other mouse, pushing the other mouse, approaching the other mouse head-on and following the other mouse. After these interactions mice were housed singly for the duration of the behavioral testing schedule.

#### Elevated plus maze

On PN22, each mouse was tested on the elevated plus maze as previously described [42]. Behavior was recorded for 10 minutes. The total time spent in the closed and open arms and the numbers of crosses through the middle were scored. Time spent in the middle of the maze was calculated based on the total duration of the test less the time in the two arms. The open arm was subdivided into proximal and distal halves and time in each was recorded.

#### Social preference tests

On PN24, mice were habituated to a testing room for one hour then placed into the center section of a three-chambered Plexiglas box (76.2 cm × 26.67 cm × 17.78 cm), divided by black Plexiglas walls and backed by black Plexiglas so that the center section was darkened on 3 sides with 2 openings leading to the outer chambers each containing a small metal cylinder with a round top (10.16 cm diam. × 13.97 cm) and vertical bars (spaced 1 cm apart), hereafter referred to as a “jail cell”. Mice were habituated to the test box in the center section with both doors closed for 10 minutes. After 10 minutes the doors were opened and the mouse was allowed to freely explore all three chambers for an additional 10 minutes. Mice were once again closed into the center section and a novel adult male was randomly placed in one of the jail cells. The use of an adult male is based on work by others in the field [43], [44]. The doors were opened to allow mice to explore all three chambers and data were collected for 10 minutes. The time spent in each chamber, the time spent sniffing each jail cell, and the numbers of entries into each side were all scored by an observer blind to sex and treatment group (adapted from [43], [44] and described in detail [16].

### Quantitative real time PCR

Total RNA was isolated from the brain tissue of male and female embryos (E18.5; n = 5–6/group) from dams given control or BPA-supplemented diet. cDNA was generated from 500 ng of total RNA by reverse transcription with iScript cDNA kit (Bio-Rad). Real time PCR was performed using the iCycler iQ™ System (Bio-Rad) according to the manufacturer's instructions for TaqMan and SYBR Green based detection. Samples were run in quadruplicates in either one or two plates. The average of the four replicates was used for data analysis. TaqMan probes for *Esr1, Esr2, Gper, Oxtr* and *B2M* (control gene) were obtained from Applied Biosystems (Carlsbad, CA). SYBR Green primers were designed for *Gria1, Grin2a, Grin2b, Dnmt1, Dnmt3a, Dnmt3b, Slc1a1* and *ß-actin* (control gene). Primer sequences for these genes can be found in Table 1. Quantification of candidate gene expression levels was calculated based on the threshold cycle (C_t_) for each well using the provided software and normalized to B2M for TaqMan assays and *ß*-actin for SYBR Green assays as endogenous controls.

**Table 1:** Primer Sequences for SYBR Green qPCR assays.

Primer	Sequence	Primer Efficiency
*ßactin* Forward	GCCACCAGTTCGCCATGGAT	103%
*ß actin* Reverse	TCTGGGCCTCGTCACCCACATA	
*Dnmt1* Forward	CCGCAGGCGGCTCAAAGACTT	99%
*Dnmt1* Reverse	GCTCCCGTTGGCGGGACAAC	
*Dnmt3a* Reverse	CTGGAAGGTGAGTCTTGGCA	89%
*Dnmt3a* Forward	GAGGGAACTGAGACCCCAC	
*Dnmt3b* Forward	AGCGGGTATGAGGAGTGCAT	103%
*Dnmt3b* Reverse	GGGAGCATCCTTCGTGTCTG	
*Gria1* Forward	TCCACTAGACCACCATCCCTTTTGT	91%
*Gria1* Reverse	ACAGAGCCTGCAAACCATGGGT	
*Grin2a* Forward	TGGTATTGCCGGCCCTTCTGGT	85%
*Grin2a* Reverse	TCACGTCGTGGCTGTGACCCA	
*Grin2b* Forward	TTCTATCCCCGGCATCCAGCG	110%
*Grin2b* Reverse	CGTGGAGCGTGGTCATTCCCA	
*Slc1a1* Forward	ATGTCAATGGGGGCTTCGCGG	100%
*Slc1a1* Reverse	AGATGGCTCCTGTGGAGACGCC	

### Statistical analysis

All data were analyzed using NCSS (2001). For behavioral data, we used two-way ANOVA with sex and diet as factors. Significant results were assessed by Fisher Exact post-hoc tests that adjust significance levels to take multiple comparisons into account. For gene expression data, normalized gene expression was calculated using the delta-delta Ct method [45]. We used two-way ANOVA with sex and diet as factors and significant results were assessed by Fisher's post hoc tests with Bonferroni's corrections for multiple comparisons.

## Results

### Plasma levels of BPA comparable to human exposure levels

Supplementing phytoestrogen-free chow with a low dose of BPA (1.25 mg per kg diet), increased blood BPA levels in gestating dams. Serum BPA levels in pregnant dams on control phytoestrogen-free diet was barely detectible at 0.099±0.014 ng/ml (n = 3). However, serum BPA levels in dams fed 1.25 mg/kg BPA supplemented phytoestrogen-free diet were four fold higher at 0.43±0.002 ng/ml (n = 2). Thus this dose produces BPA exposure well within the range detected in human maternal blood, 0.3 to 18.9 ng/ml [2].

### Gestational BPA exposure affects social interactions particularly in females

**Figure 1:**
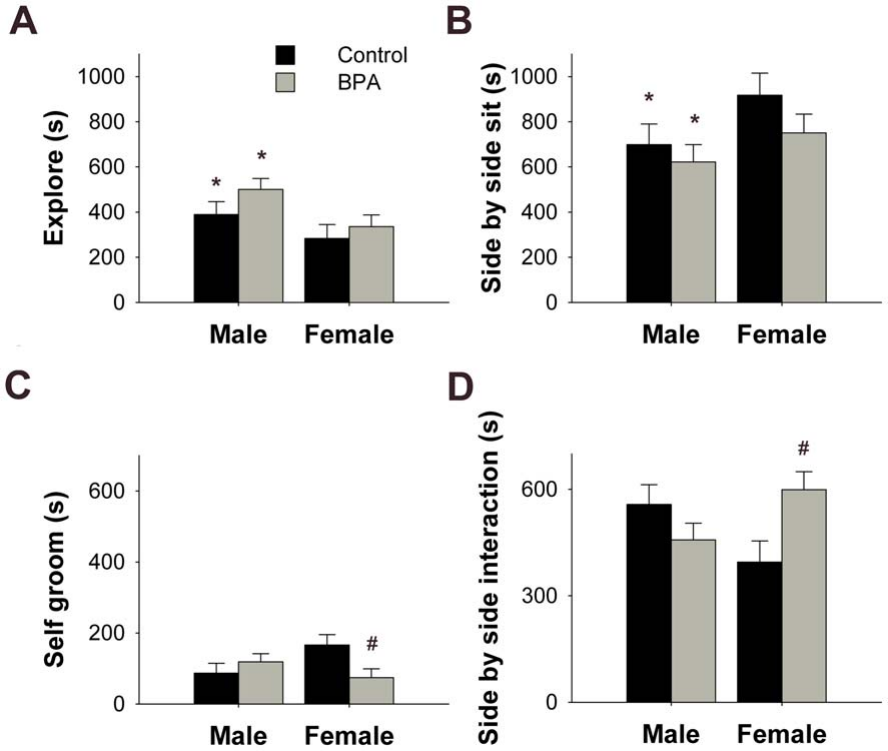
Mean +/- SEM behaviors displayed during a reciprocal social interaction task. Juvenile male and female mice were exposed to control (phytoestrogen-free chow, black histograms, n = 15 males and n = 13 females) or BPA supplemented chow (gray histograms, n = 21 males and n = 18 females) during gestation. **A**) Total amount of time in seconds spent exploring the cage. **B**) Duration of side by side sitting (seconds). **C**) Amount of time spent self grooming (seconds). **D**) Duration of side by side interactions (seconds). * Sex effect; males are significantly different from females, p<0.05. ^#^ Significantly different from control females, p<0.05.

Several of the behaviors observed were sexually dimorphic. In general, males spent more time engaging in non-social behaviors, while females spent more time in social contact. Males spent more time exploring the cage and sitting alone (F_1,63_ = 6.8, 4.91, respectively p<0.05 at least) as compared to females. In contrast, females spent more time sitting side-by-side than did males (F_1,63_ = 3.92, p = 0.05, Figure 1 and Table 2).

**Table 2:** Mean +/− SEM time spent (in seconds) or frequencies of different types of behavior in a 30- minute social interaction test.

	Control Male n = 15	BPA Male n = 21	Control Female n = 13	BPA Female n = 18
**Groom partner (s)**	31.2±9.9	17.8±9.8	27.6±13.0	20.2±4.5
**Side by Side Sitting (s)**	698.9±78.0	621.8±78.6	917.5±110.3 !	750.1±84.7 !
**Side by Side behaviors other than grooming (s)**	557.1± 40.5	457.1±45.9	394.7± 53.3	599.0±63.4 *
**Self Groom (s)**	87.1±17.6	118.5±20.8	166.0±49.4	74.1±16.7 *
**Sit Alone (s)**	12.0±12.0 !	66.1±26.1 !	10.4±9.3	0.0±0.0
**Explore (s)**	389.2±49.7 !	500.5±48.9 !	283.2±79.8	335.5±44.5
**Anogenital Sniff**	0.1±0.1	0.3±0.1	0.1±0.1	0.2±0.1
**Nose-Nose Sniff**	1.9±0.4	3.1±0.6 #	1.7±0.5	2.7±0.6 #
**Crawl**	1.1±0.3	0.9±0.3	0.7±0.3	1.2±0.4
**Follow other animal**	0.67±0.23	0.33±0.12	0.23±0.12	0.89±0.31 *
**Push**	0.2±0.1	0.5±0.2	0.2±0.1	0.2±0.1
**Approach**	3.2±0.5	4.2±0.7 #	2.8±0.7	4.9±0.9 #

! Significant effect of sex p<0.05,

* Significantly different from the same sex control p<0.05,

# Significant effect of diet p<0.05.

Interactions between diet and sex were noted for several other behaviors and in all cases the interactions were caused by differences between BPA and control females. Side-by-side interactions that did not include grooming (F_1,63_ = 8.13, p<0.006) were exhibited for a longer duration by BPA exposed females as compared to the control females (p<0.05). An example of this behavior is sitting together in a corner and sifting through the bedding. An interaction in the numbers of times one mouse followed its partner was found (F_1,63_ = 5.01, p<0.03). Again, BPA treated females performed following more often than control females (p<0.05). Lastly, self-grooming also showed an interaction (F_1,63_ = 5.51, p<0.02) caused by differences between BPA and control females. Of interest this non-social behavior was exhibited for a longer time by control than BPA treated females. In general these results suggest that BPA treated females are more interactive than controls.

Diet affected the frequencies of nose-to-nose contacts, and numbers of approaches. For both of these contact behaviors mice exposed to BPA displayed more than controls (F_1,63_ = 3.99, 3.82 respectively; p<0.05). No differences were noticed in the duration of time spent grooming the partner, ano-genital sniffing, crawling and pushing. These behaviors were also exhibited in very low amounts.

### BPA did not affect social preferences or anxiety in juveniles

Social preference in this task was sexually dimorphic in the juvenile mice. Investigation of an adult C57BL/6J male was greater in males as compared with females (F_1,66_ = 4.43, p<0.05) and no activity differences were detected as the number of times that mice crossed between the three sections of the test box did not vary by group (Table 3). Gestational exposure to BPA did not affect social preference for the stimulus animal, nor did it alter the sexual dimorphism observed in controls. Anxiety, assessed in the elevated plus maze, did not differ between the groups. The time spent in the open arms, closed arms and the number of crosses between arms were similar in all groups (Table 4).

**Table 3:** Mean +/- SEM time spent in one of three chambers in a 3-chambered preference test with an adult mouse stimulus animal.

	Sociability Score	Time with Mouse (s)	Time in Empty (s)	Time in Center (s)
**Control Male** n = 15	140.5±40.7 *	321.0±26.5 *	180.5±18.2	98.4±20.3
**BPA Male** n = 21	51.6±44.0 *	252.2±28.5 *	200.6±19.0	143.7±20.8
**Control Female** n = 13	15.2±55.1	245.4±29.3	230.2±29.9	124.3±21.3
**BPA Female** n = 18	-0.8±30.8	212.1±21.9	213.0±20.2	177.2±30.8

Sociability Score  =  time with mouse minus time in empty.

*Effect of sex, p<0.05.

**Table 4:** Mean +/− SEM time spent in different sections of the elevated plus maze during a 10 minute test.

	Open (s)	Distal Open (s)	Closed (s)	Center (s)	# Crosses
**Control Male** n = 15	78.9±5	36.6±3	399.8±12	121.4±10	23.8±2
**BPA Male** n = 21	75.5±6	34.4±4	407.7±12	116.8±9	23.1±1
**Control Female** n = 13	80.0±15	33.5±8	410.9±23	109.1±11	22.8±3
**BPA Female** n = 18	77.9±8	33.9±4	409.8±15	112.2±9	22.7±2

No significant differences were found in any anxiety phenotype in juvenile mice exposed to BPA.

### BPA exposure affects gene expression

**Figure 2:**
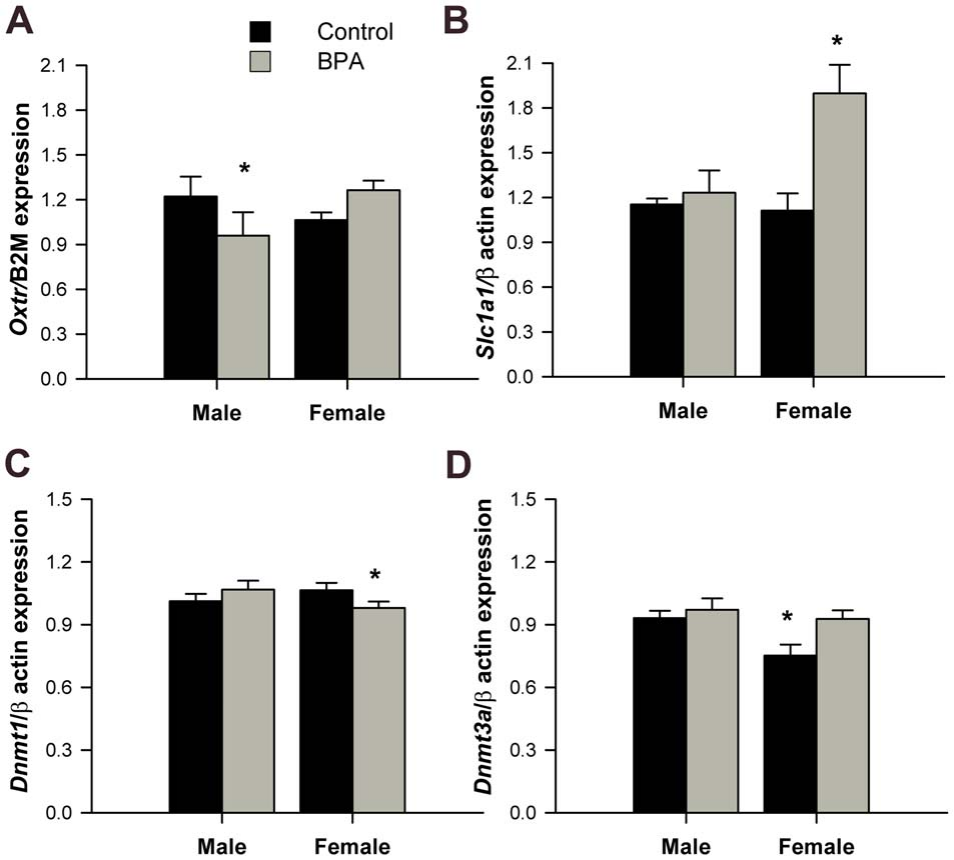
Mean +/- SEM mRNA expression measured with qPCR in embryonic day 18.5 brain relative to control males and normalized to beta-2 microglobulin or beta-actin (n = 5–6/group). Black bars indicate groups exposed to control, phytoestrogen-free diet. Gray bars indicate groups exposed to BPA during gestation. A) Oxytocin receptor (*Oxtr*). B) Neuronal glutamate transporter (*Scl1a1*). C) DNA methlytransferase 1 (*Dnmt1*). D) DNA methyltransferase 3a (*Dnmt3a*). ^*^Significantly different from all other groups, p<0.05.

Two very interesting candidate genes were affected by diet and sex. Expression of the glutamate transporter, *Scl1a1,* was sexually dimorphic (F_1,18_ = 4.58, p<0.05) with females having higher mRNA than males, and a significant interaction between diet and sex (F_1,18_ = 5.84, p<0.04, Figure 2) was noted. The interaction was caused by the female BPA group, which had higher expression levels than any other groups. Expression of the oxytocin receptor indicated a trend for an interaction (F_1,18_ = 4.14, p = 0.06; Figure 2). This trend was caused by the BPA male group, which had significantly less *Oxtr* mRNA than any other group (p<0.05, Figure 2).

BPA can bind estrogen receptors and also affects methylation of DNA. We examined expression of three estrogen receptor genes, *Esr1*, *Esr2*, and *Gper*; none of which was impacted by diet or sex (Table 5). We also quantified mRNA for the three DNA methytransferase genes; *Dnmt1*, *Dnmt3a* and *Dnmt3b*. Both *Dnmt1* and *Dnmt3a* genes were responsive to diet and/or sex. BPA exposure increased expression of *Dnmt3a* as main effects of diet were observed (F_1,18_ = 5.13, p<0.04). At embryonic day 18.5, *Dnmt3a* expression was also sexually dimorphic (F_1,18_ = 5.49, p<0.04) with males having more mRNA than females. *Dnmt1* expression exhibited a trend for interaction between sex and diet (F_1,18_ = 3.68, p<0.07) produced by a lowest amount of mRNA in brains of BPA exposed females as compared with all other groups (p<0.05, Figure 2). Expression of *GluR1*, *Grin2a* and *Grin2b* were not influenced by sex or diet (Table 5).

**Table 5:** Mean +/- SEM for gene expression in E18.5 brains relative to control males and normalized to beta-2-microglobulin or beta-actin.

	*Esr1*	*Esr2*	*Gper*	*Dnmt3b*	*Gria1*	*Grin2a*	*Grin2b*
**Control Male**	0.76±0.10	1.14±0.11	1.06±0.02	1.05±0.06	0.99±0.05	0.95±0.09	1.01±0.13
**BPA Male**	0.78±0.04	0.89±0.16	0.95±0.08	1.03±0.07	0.95±0.04	0.85±0.04	0.98±0.09
**Control Female**	0.71±0.02	1.15±0.07	1.15±0.04	0.98±0.03	0.95±0.02	0.71±0.05	0.94±0.05
**BPA Female**	0.77±0.03	1.06±0.05	1.11±0.06	0.96±0.05	1.04±0.07	0.82±0.04	1.11±0.06

*Esr1*  =  estrogen receptor alpha, *Esr2*  =  estrogen receptor beta, *Gper*  =  G protein-coupled estrogen receptor, *Dnmt3b*  =  DNA methyltransferase 3b, *Gria1*  =  AMPA ionotropic glutamate receptor, *Grin2a*  =  NMDA receptor subunit 2a, *Grin2b*  =  NMDA receptor subunit 2b.

These genes were not significantly altered by BPA exposure or sex. N = 5–6 brains in each group.

## Discussion

Our goal was to assess the effects of BPA on several behaviors in juvenile mice using a very low dose of BPA, within a range comparable to humans. We also limited exposure to the gestational period when most neural development occurs. We did so not only to limit the exposure time but also to remove the complicating effects that BPA may have on maternal behavior [16], [39]. Thus by fostering all pups to dams on control diet, we control for the potential contribution of differences in maternal care. Needless to say fostering introduces a new set of issues, which we controlled for here by fostering all the animals used in the experiments. Incorporating BPA in chow we produced tonic levels in mouse dams comparable to humans and we assume these were also achieved in the embryos. BPA readily crosses the placenta [46], [47], exposing fetal tissues to concentrations close to those in maternal placenta [48]. Furthermore, in its inactivate conjugated form, BPA, can be unconjugated and re-activated in the fetus [47]. Thus, our dosing method is a highly relevant for studying the effects of gestational BPA exposure and subsequent behavioral and genomic profiles. Using a dose well below the LOAEL, given only during gestation, we detected several effects of BPA in diet on juvenile social behaviors as well as embryonic gene expression in whole brain, which may underlie the observed behavioral effects.

Our most striking findings were in females exposed to BPA during a juvenile dyadic social interaction task. This is an “open-ended” task used to identify social, non-social, play-soliciting and investigative behaviors between pairs of mice of the same sex and age [40], [41]. BPA exposed females displayed increased social interactions. They engaged in more side-by-side interactions and followed each other more than pairs of control females. They also displayed less self grooming, a non-social behavior. Moreover, in two other measures of partner interactions; nose-to-nose investigations and approaches, BPA treated animals were more gregarious than controls. Together, these results suggest that gestational exposure to BPA in female mice increases social investigations and interactions. BPA exposure, particularly when restricted to critical brain developmental periods, alters the sexually dimorphic brain and in certain behaviors, females appear to be more sensitive to the effects of BPA. For example, BPA exposure decreased the number of tyrosine hydroxylase (TH) positive cells in females, eliminating the sexual dimorphism in the anteroventral periventricular region of the hypothalamus [11]. Females exposed to BPA during the period of brain sexual differentiation were less reactive and explored a novel environment less than control females [49]. Amphetamine-induced conditioned place preference is also disrupted in female mice exposed to BPA during gestation while males displayed no change in place preference [50]. In rats, BPA exposure at various time points during gestation and after birth affects juvenile social behaviors. In juvenile females, BPA increased both non-social and social investigation [15]. When tested with males, juvenile females exposed to BPA exhibited reduced play and social grooming. In a study comparing male and female rat juvenile social behavior, BPA reduced social interest in both sexes but a lower dose exposure increased social approach and interactions in females [17]. Our data add to these conclusions showing that in juvenile female mice, even at very low doses, BPA *in utero* increases their display of social interactions.

Interestingly in the present study, juvenile males were less social than females, exhibiting higher levels of cage exploration and sitting alone than did females. Females on the other hand spent more time in side-by-side sitting than did males. Sex differences have been reported in normal C57BL/6J juvenile mice tested in the same manner reported here [41]. In that study, males engaged in more social interactions, and females performed more play soliciting. In CD-1 mice, on several tasks, BPA exposure reduced the sex differences observed in controls [49]. Several procedural differences between these studies likely cause the variation in results. In the present studies, all mice were reared on a phytoestrogen-free chow, as opposed to normal rodent chow containing phytoestrogens. Phytoestrogens in chow can enhance sex differences in the brain and in behavior [51], [52], [53], [54], [55]. Second, mice in the present study were fostered at birth and we cannot exclude the possibility that fostering may have as yet unidentified effects which alter the display of sex differences. The few studies directly assessing the effects of fostering on maternal rearing behavior have conflicting results [56], [57] but raise the possibility that C57BL/6J dams may lick and/or groom the fostered pups more than their biological pups.

In a preference task, juvenile males spent more than 50% of their time in the chamber with an adult male mouse instead of the empty chamber. Exposure to BPA at the current dose did not significantly alter these behaviors. However a 40-fold higher BPA dose decreased male social preference in the identical task [16]. Likewise we did not find any effects of sex or BPA on elevated plus maze behavior in the current study. Yet again, exposure to the 40-fold higher BPA dose decreased time in the middle, increased time in the closed arm, and tended to decrease time in the outer most portion of the open arm [16], indicating that BPA at this high dose increased anxiety-related behaviors in the plus maze. These data demonstrate that different juvenile behaviors are affected by different doses of BPA.

There are several potential mechanisms underlying the sex-specific behavioral responses to BPA exposure. Males, but not females, experience a surge of testosterone (and estradiol) during the last few days of gestation [52]. Thus endogenous estrogens may out-compete BPA, for example, for estrogen receptor binding sites in males but not in neonatal females that are not experiencing an endogenous hormone surge. Another possibility is that genes on sex chromosomes may interact with BPA [58]. Our embryonic gene expression results tend to support the first potential mechanism. We assayed expression levels of estrogen receptor (ER) genes in E18.5 brains to ask if BPA would affect their expression. We selected this embryonic age because it is when brain sexual differentiation occurs under the influence of sex differences in blood levels of testosterone and brain levels of estrogens [21], [52]. We hypothesized that if BPA acted via one or more ER it might down regulate this receptor, perhaps in a manner similar to the natural ligand. However, we found no changes in any of the ER or putative ER genes based on BPA exposure. This does not mean that BPA did not act as a direct ligand for one or more of the ERs. In fact it is likely that BPA did influence ERα since the oxytocin receptor is an ERα target gene and we noted changes in *Oxtr* mRNA [59], [60]. In neonatal cortex, estradiol hypermethylates ERα [61] and perhaps the same thing occurs here, but, given that we assayed mRNA in whole brain and ERα expression is not global we may have missed BPA induced changes in expression.

Another mode of BPA action is via direct changes DNA methylation [29]. We assayed the three known DNA methyltransferases, enzymes responsible for the deposition of methyl groups onto cytosines when followed by a guanine. *Dnmt3a*, a gene responsible for *de novo* methylation, was altered by BPA exposure wherein the sex difference in control animals (male>females) was not present in BPA exposed brains because BPA increased *Dnmt3a* expression in females. *Dnmt3a* has been implicated in rewarding behavior and neuronal plasticity in the adult mouse accumbens [62]. *Dnmt1* is the most abundant DNA methyltransferase and is believed to be responsible for the maintenance of DNA methylation. Interestingly, *Dnmt1* gene was decreased by BPA in female embryo brains and we speculate that the drop in this gene might be related to the higher levels of *Slc1a1* in these same brains.

In male mice, exposed to low doses of BPA that are probably comparable to ours, mRNA for ERβ and several of the NMDA receptors were decreased in hippocampus at day 21 and 56 [33]. In addition in male rats, LTD and LTP were disrupted by a dose of BPA higher than ours [63]. These effects were attributed to functional alterations in dopamine, glutamatergic, and metabotropic glutamate receptors, but in our study none of the genes we assayed, related to the later two pathways, were affected by BPA. Of course, our treatment period was shorter than theirs; in both studies dams were placed on the BPA diet around E7 and pups stayed on the diet through weaning. Moreover, we examined mRNA at a different time point, but none of the three genes (*Gria1, Grin2a, Grin2b* and *Esr2*) were affected by diet in our study.

Despite the lack of change in NMDA and AMPA receptors, the largest gene expression effect was noted for *Slc1a1*, one of the glutamate transporter genes, and interestingly its expression was elevated in brains of females exposed to BPA. *Slc1a1* is found throughout the cortex, hippocampus and basal ganglia [64] and functions to buffer local glutamate at excitatory synaptic connections [65]. Behavioral characterization of the *Slc1a1* null mouse is not extensive but with age these animals appear to have impairments in self-grooming and spatial learning [66]. In humans single nucleotide polymorphisms (SNPs) within this gene have been associated with repetitive behaviors and anxiety in children with ASD [67], mental retardation [68] and obsessive compulsive disorder [69], [70], [71], [72]. The last candidate gene we examined, the oxytocin receptor, has long been associated with the display of social behavior in rodents [34]. A polymorphic region of the oxytocin receptor (OXTR) in humans has been associated with empathy and stress reactivity [73]. Additionally, many have speculated that OXTR is involved with autism spectrum disorders. Two genome wide association studies in autistics patients show linkage associations to chromosomal region 3p25.3, which contains OXTR [74], [75]. Together, our findings that *in utero* BPA exposure alters expression of *Oxtr* and *Slc1a1* suggest a potential mechanism through which early life exposure to BPA can alter normal signaling in the brain and effect adult neurological disorders such as the pathophysiologies associated with ASD, obsessive compulsive disorder and mental retardation.
